# Understanding Mechanisms of Salinity Tolerance in Barley by Proteomic and Biochemical Analysis of Near-Isogenic Lines

**DOI:** 10.3390/ijms21041516

**Published:** 2020-02-22

**Authors:** Juan Zhu, Yun Fan, Sergey Shabala, Chengdao Li, Chao Lv, Baojian Guo, Rugen Xu, Meixue Zhou

**Affiliations:** 1 Key Laboratory of Plant Functional Genomics of the Ministry of Education / Jiangsu Key Laboratory of Crop Genomics and Molecular Breeding/ Jiangsu Co-Innovation Center for Modern Production Technology of Grain Crops/ Institutes of Agricultural Science and Technology Development, Yangzhou University, Yangzhou 225009, Chinaclv@yzu.edu.cn (C.L.); bjguo@yzu.edu.cn (B.G.); 2 Tasmanian Institute of Agriculture, University of Tasmania, Private Bag 1375, Prospect, TAS 7250, Australia; yun.fan@utas.edu.au (Y.F.); sergey.shabala@utas.edu.au (S.S.); 3 International Research Centre for Environmental Membrane Biology, Foshan University, Foshan 528000, China; 4 Western Barley Genetics Alliance, School of Veterinary and Life Sciences, Murdoch University, Murdoch, WA 6150, Australia; c.li@murdoch.edu.au

**Keywords:** Barley (*Hordeum vulgare* L.), near-isogenic lines, salinity tolerance, photosynthesis, ROS scavenging, ATP synthase

## Abstract

Salt stress is one of the major environmental factors impairing crop production. In our previous study, we identified a major QTL for salinity tolerance on chromosome 2H on barley (*Hordeum vulgare* L.). For further investigation of the mechanisms responsible for this QTL, two pairs of near-isogenic lines (NILs) differing in this QTL were developed. Sensitive NILs (N33 and N53) showed more severe damage after exposure to 300 mM NaCl than tolerant ones (T46 and T66). Both tolerant NILs maintained significantly lower Na^+^ content in leaves and much higher K^+^ content in the roots than sensitive lines under salt conditions, thus indicating the presence of a more optimal Na^+^/K^+^ ratio in plant tissues. Salinity stress caused significant accumulation of H_2_O_2_, MDA, and proline in salinity-sensitive NILs, and a greater enhancement in antioxidant enzymatic activities at one specific time or tissues in tolerant lines. One pair of NILs (N33 and T46) were used for proteomic studies using two-dimensional gel electrophoresis. A total of 53 and 51 differentially expressed proteins were identified through tandem mass spectrometry analysis in the leaves and roots, respectively. Proteins which are associated with photosynthesis, reactive oxygen species (ROS) scavenging, and ATP synthase were found to be specifically upregulated in the tolerant NIL. Proteins identified in this study can serve as a useful resource with which to explore novel candidate genes for salinity tolerance in barley.

## 1. Introduction

Soil salinity, one of the major abiotic stresses, has become a serious issue limiting agricultural production and threatening environmental health and economic welfare [1]. It is estimated that approximately 20% of the world’s cultivated land and nearly half of all irrigated land are affected by salinity [2]. The response of plants to salinity stress presents complex quantitative traits that are affected by multiple environmental factors, involving complex physiological and molecular mechanisms [3]. Despite extensive and numerous studies having been conducted over the past few decades on the responses and mechanisms of salinity tolerance in plants, little progress has been made to date in developing high-yielding, salt-tolerant genotypes because of the genetic and physiological complexity of salinity tolerance [4] and a lack of reliable screening methods [5].

Maintaining optimal respiratory and photosynthesis processes is crucial for salinity tolerance. Osmotic stress and high accumulation of toxic Na^+^ in cytoplasm induce stomatal closure which causes a strong imbalance between light capture and energy utilization, reduces the photosynthetic rate, impairs the bioenergetic processes of photosynthesis, and leads to the formation of reactive oxygen species (ROS), such as superoxide radical (O_2_^•−^), hydroxyl radical (OH^•^), singlet oxygen (^1^O_2_), and hydrogen peroxide (H_2_O_2_) [6,7]. In turn, the accumulation of ROS can further activate GORK (depolarization-activated outward rectifying K^+^ channel) and ROS-activated NSCC (nonselective cation channels), which induces a rapid loss of K^+^ from the cytosol and interferes Na^+^ /K^+^ ratio [8,9,10]. The effect of salinity on mitochondrial respiration has not been fully understood, with differing reports showing both increased or decreased respiration in response to salt stress [11]. A high respiration rate produces more ATP, which provides vital energy for defense against salinity stress, such as osmotic adjustment, ion exclusion, and compartmentation [12]. However, high respiration rates lead to excessive carbon being consumed by respiration, rather than in the synthesis of new tissue [13], and more ROS generation because of the overreduction of the electron transport in mitochondria [14].

Salt-tolerant plants have evolved various mechanisms that function in coordination to alleviate osmotic pressure and maintain ionic homeostasis in cells. It is essential for plant growth under salt stress to maintain ionic homeostasis through the regulation of ion uptake and compartmentalization [15,16]. Plants can reduce Na^+^ accumulation through the following mechanisms to minimize plant salt damage: (1) reduced Na^+^ uptake by plant roots; (2) reduced Na^+^ loading into the xylem; (3) enhanced capacity of Na^+^ re-translocation back into roots; (4) compartmentalization of Na^+^ into vacuole; and (5) salt secretion on the leave surface. The regulation of Na^+^ homeostasis in plants involves an orchestrated operation of various Na^+^ transporters or channels such as cyclic-nucleotide gated channels (CNGCs) [17], glutamate receptors (GLRs) [18], nonselective cation channels (NSCCs) [19], high affinity (Na^+^)K^+^ HKT transporters [19], plasma membrane [20], and tonoplast-based [21] Na^+^/H^+^ transporters (SOS1 and NHX, respectively). An optimal cytosolic K^+^/Na^+^ ratio can also be achieved by the efficient regulation of K^+^ homeostasis [22]. In *Arabidopsis*, 35 K^+^-selective transporting systems from five major groups are known, i.e., Trk/HKT transporters, KUP/HAK/KT transporters, KCO (2P/4TM) K^+^ channels, Shaker-type (1P/6TM) K^+^ channels, and K^+^/H^+^ antiporter homologs [23]. Salinity tolerance also implies the accumulation of compatible solutes in the cytosol. These may have important functions in osmotic adjustment, the protection of enzyme and protein structures, the stabilization of photosystem II complexes, the maintenance of cell membrane integrity, the removal of reactive oxygen species (ROS), and the reduction of K^+^ [24]. Plants have also evolved complex ROS defense mechanisms, including enzymatic and nonenzymatic pathways to scavenge excessive ROS, thereby controlling ROS at an optimal level for signaling [25]. Converting the superoxide (O_2_^•−^) to hydrogen peroxide (H_2_O_2_) by SOD is considered the initial and first vital step against oxidative stress in plants. Following the dismutation reaction, hydrogen peroxide can be catalyzed by CAT, POX, and APX into water and oxygen.

Barley is one of the most salinity-tolerant crops [7]. It is an excellent model crop for studies on the mechanisms and inheritance of salinity tolerance and for developing tools to improve salt tolerance in cereals. Investigating the physiology and molecular mechanisms could provide global insight into the characteristics of salinity responses in plants and help to identify key genes involved in barley salinity tolerance. In our previous studies, one major QTL, *QSl.TxNn.2H*, associated with salinity tolerance, was identified [26]. In the present work, we have undertaken a biochemical and proteomic analysis of two pairs of near-isogenic lines (NILs) that are almost genetically identical, except for the target region containing *QSl.TxNn.2H*. The major objectives of this work were: (1) to evaluate the effects of *QSl.TxNn.2H* on the biochemical characteristics of the barley plants; (2) to understand the mechanisms of salinity tolerance by the combination of biochemical and proteomic analyses; and (3) to explore novel candidate genes for salinity stress tolerance in barley.

## 2. Results

### 2.1. Near Isogenic Lines Exhibited Contrasting Salinity Tolerance

**Figure 1:**
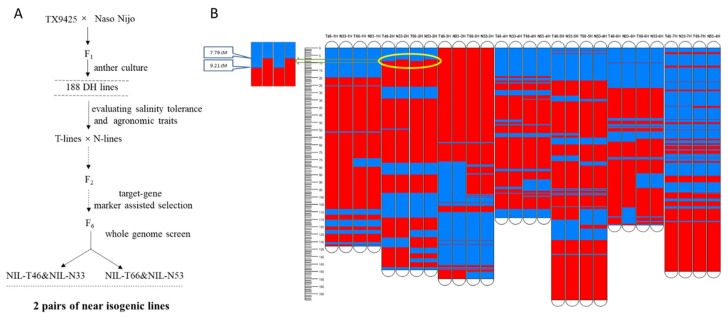
Construction of two pairs of NILs. (**A**): Strategy for developing NILs. (**B**): Comparison between genotypes of near isogenic (Pair 1: T46/N33; Pair2: T66/N53). Red: Naso Nijo backgrounds; blue: TX9425 backgrounds. Yellow circle: the major difference on 2H at the position of 6.6-9.4 cM for two pair of NILs.

**Figure 2:**
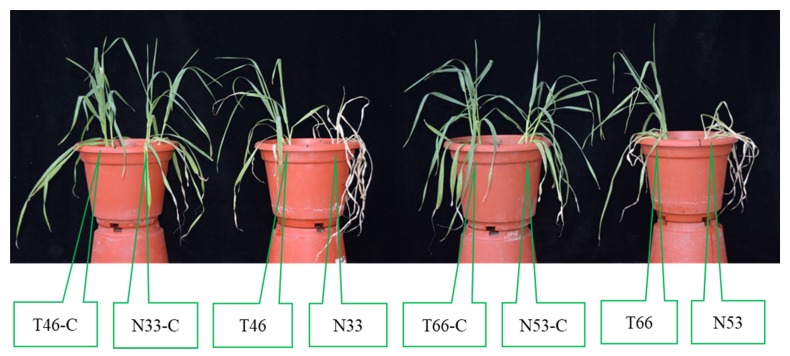
Performance of NILs treated with 300 mM NaCl for 10 days. Control plants (C) were treated with tap water.

Two pairs of NILs (N33 and T46, N53 and T66) were constructed (Figure 1A) which had almost identical genetic backgrounds, except for the major salinity tolerance QTL region (2.8 cM) on chromosome 2H (Figure 1B). Two pairs of NILs showed similar morphologies under control conditions but contrasting salinity tolerance under salt treatment. T46 and T66 showed salt tolerance with no apparent symptoms of leaf chlorosis or wilting, while N33 and N53 showed salt sensitivity with severe chlorosis and low survival (Figure 2).

### 2.2. Effect of Salinity on Na^+^ and K^+^ Contents and Na^+^/ K^+^ Ratios

**Figure 3:**
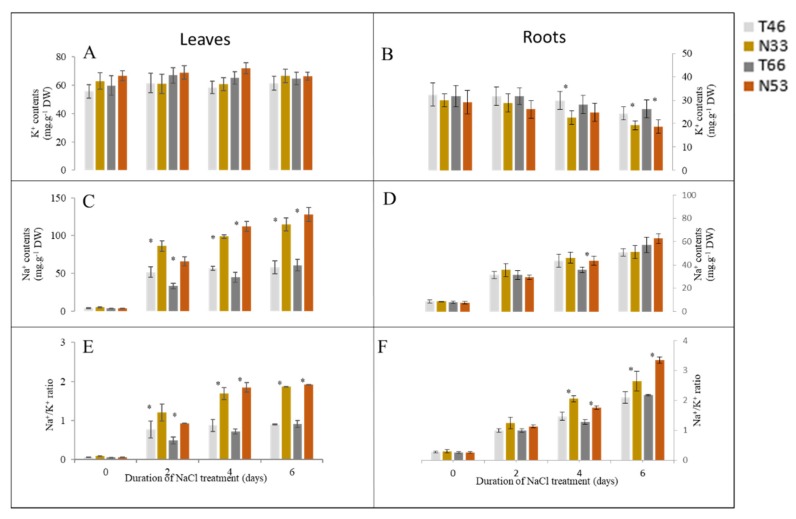
Comparison of Na^+^ content (**C**,**D**), K^+^ content (**A**,**B**) and Na^+^/K^+^ ratio in (**E**,**F**) leaves (**A**,**C**,**E**) and roots (B,D,F) of two pairs of NILs at 0, 2, 4, 6 days after 300 mM NaCl. * indicates significant differences among NILs (*p* < 0.05, Student’s *t*-test).

In leaves, NILs showed a time-dependent, continuous increase in Na^+^ contents and Na^+^/K^+^ ratio when grown under saline conditions, with sensitive lines showing significantly higher Na^+^ contents and Na^+^/K^+^ ratios. The amount of accumulated Na^+^ in sensitive NILs was 2-fold more compared with tolerant NILs after 10 d salinity treatment (Figure 3C,E). No significant changes were observed in the leaf K^+^ content after salinity stress over 10 days of stress exposure (Figure 3A).

In roots, salinity exposure also resulted in an increased Na^+^ accumulation. However, no significant (at *p* < 0.05) difference was found between contrasting NILs for this trait (Figure 3D). Salinity treatment also resulted in a progressive decline in root K^+^ content in both NILs. This decline was much more pronounced in salt-sensitive lines (significant at *p* < 0.05; Figure 3B).

### 2.3. The Accumulation of H_2_O_2_, MDA, and Proline After Salinity Treatment

**Figure 4:**
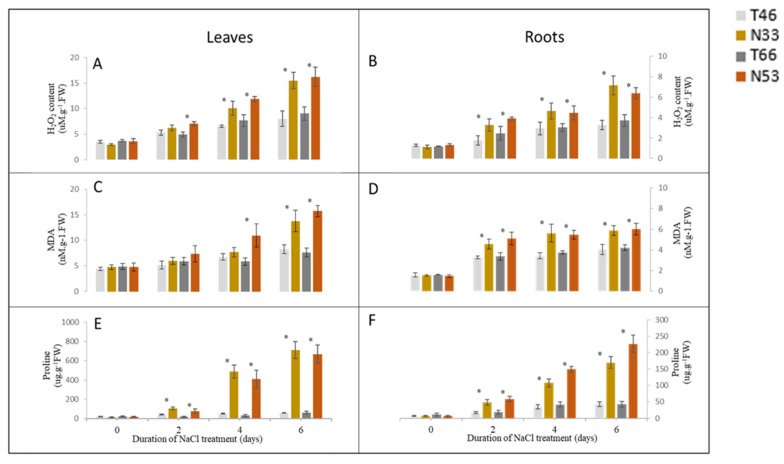
Comparison of_,_ H_2_O_2,_ MDA and proline contents in leaves (**A**,**C**,**E**) and roots (**B**,**D**,**F**) of two pairs of NILs at 0, 2, 4, and 6 days after 300 mM NaCl treatment. * indicates significant differences among NILs (*p* < 0.05, Student’s *t*-test).

Hydrogen Peroxide (H_2_O_2_) is a metabolic by-product of reactive oxygen that serves as an indicator of the ability to remove ROS, while the amount of MDA reflects the degree of lipid peroxidation and shows the degree of cell damage indirectly. Changes of H_2_O_2_ and MDA contents in leaves and roots in response to NaCl treatments are shown in Figure 4. The contents of H_2_O_2_ and MDA increased in both the tolerant and sensitive lines under salt stress. However, compared with the tolerant lines, both the leaves and roots in the sensitive lines accumulated greater H_2_O_2_ and MDA (Figure 4A–D).

The proline contents during salt stress revealed a stark contrast between the tolerant and sensitive lines. After 6 days of treatment, the proline contents in the salt-sensitive lines were remarkably higher than in salt-tolerant lines, with a more than 10-fold increase in leaves and 4-fold increase in roots (Figure 4E,F).

### 2.4. Changes in Activities of Antioxidant (AO) Enzymes under Salinity Stress

**Figure 5:**
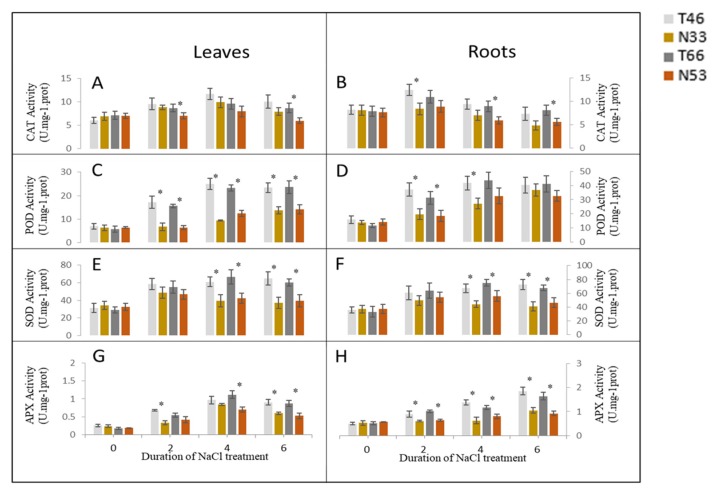
Antioxidant enzyme activities of leaves (**A**,**C**,**E**,**G**) and roots (**B**,**D**,**F**,**H**) from two pairs of NILs at 0, 2, 4, and 6 days after 300 mM NaCl treatment. * indicates significant differences among NILs (*p* < 0.05, Student’s *t*-test).

The activities of CAT, SOD, POD, and APX in leaves and roots were measured after NILs were exposed to salinity stress at 0, 2, 4, and 6 days. CAT activities increased first and then decreased in both leaves and roots of all NILs, while the tolerant line T66 showed significantly higher CAT activities than N53 at 2 and 6 days in leaves, as well as at 4 and 6 days in roots after salt treatment (Figure 5A,B). Significantly higher SOD increases were observed in tolerant lines at 4 and 6 days in both leaves and roots (Figure 5E,F). In comparison with the control, POD and APX activities increased after exposure to salinity stress in leaves and roots of all NILs with tolerant lines showing higher activities (Figure 5C,D,G,H).

### 2.5. Identification of Differentially Expressed Proteins

All differentially expressed protein spots between the control and the salinity treated N33 and T46 were excised for identification. A total of 53 (Table S1) and 51 (Table S2) differentially expressed proteins were identified, using ESI-QTOF MS and Mascot database searching, in the leaves and roots, respectively. Based on the Gene Ontology, Uniprot, MapMan, and information from the literature, these 104 proteins were involved in 11 functional categories: photosynthesis (16), stress and redox (24), protein degradation, synthesis, folding, assembly and modification (11), cell organisation and division (5), metabolism (16), glycolysis (6), oxidative pentose phosphate pathway (4), RNA transcription and binding (3), transport (4), TCA (2), development (1), and unknown (12).

**Figure 6:**
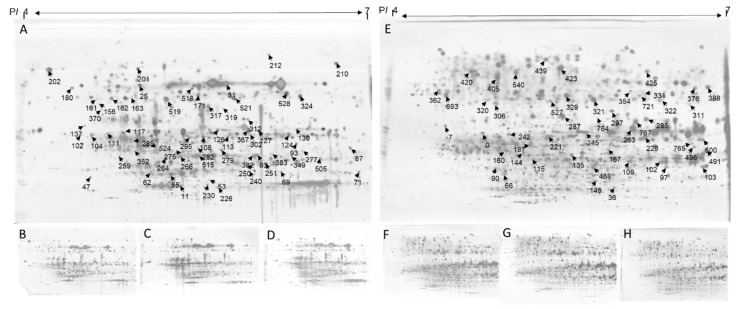
2-DE gels from leaves (**A**,**B**,**C**,**D**), roots (**E**,**F**,**G**,**H**) show the positions of spots listed in Table S1 and Table S2. Arrow indicate salt-responsive spots of which have been identified by MS. A: N33 leaves after exposure to 300 mM NaCl 4 days; B: T46 leaves after exposure to 300 mM NaCl 4 days; C: N33 leaves under control conditions; D: T46 leaves under control conditions; E: N33 roots after exposure to 300 mM NaCl 4 days; F: T46 roots after exposure to 300 mM NaCl 4 days; G: N33 roots under control conditions; H: T46 roots under control conditions.

In leaves, compared with control conditions, 18 upregulated and 2 downregulated proteins were identified following exposure to salt stress in both N33 and T46. There were 12 proteins that were upregulated in response to salt stress in salt tolerant T46, but which remained unchanged in N33. Four of these proteins were identified as functioning in photosynthesis (i.e., spots 127, 251, 266, 528). In addition, proteins related to RNA binding and transcription (spots 102, 226, 250), stress and redox (spots 319, 519), glycolysis (spot 126), and protein assembly and synthesis (spots 111, 162) were specially upregulated in T46. Eight downregulated and 13 upregulated proteins were found in N33, but no obvious changes were detected in T46. The 8 downregulated proteins were involved in photosynthesis (spots 11, 83, 104, 124), protein synthesis (spot 31) and stress and redox (spot 201). In roots, compared with control conditions, 11 upregulated and 9 downregulated proteins were identified following exposure to salt stress in both N33 and T46. One downregulated and 15 upregulated proteins were identified only in T46 and 9 downregulated and 4 upregulated proteins were identified only in N33. Two proteins showed a contrasting response to salt stress in N33 and T46. The specific upregulated proteins in T46 were mainly involved in stress and redox (spots: 187, 102, 109, 167, 228, 245, 767) and mitochondrial electron transport (spots: 287, 36, 322) (Figure 6, Table S1 and Table S2).

## 3. Discussion

### 3.1. Near Isogenic Lines With Contrasting Salinity Tolerance Poses Less Background Noise

The current knowledge of the physiology and proteomics of salinity tolerance in barley mostly relies on comparative studies of varieties with contrasting responses towards salinity [27,28,29,30,31]. NILs offer unique advantages in physiological and genetic studies, since only two isolines are involved in assessing the effect of a particular allele, and the fixed genetic background avoids the noise from other genes [32]. In this study, two pairs of NILs with contrasting salinity tolerance caused by an allele at *QSL.TxNn2H* were developed. As expected, the lines have the *QSL.TxNn2H* allele from the tolerant parents were highly salt-tolerant, which further confirmed the significance of this QTL. In a previous work of Fan et al. [33], changes in Na^+^ content, Na^+^/K^+^ ratio, proline, MDA, and AO enzyme activities in leaf were analyzed using the parents of NILs (TX9425 and Naso Nijo) under salinity stress. While some differences in AO enzyme activity responses to salinity were detected [33], changes in leaf AO enzyme activities induced by salinity had no correlation with plant grain yield or survival rate. However, in our studies, even though differences in AO enzyme activities among NILs were not stable, significantly higher AO activity was detected at each specific time or tissue in tolerant lines compared with sensitive lines. The difference between these two studies may be potentially explained by too much background noise interference in two varieties. This notion is supported by the findings that there are very big differences in AO enzyme activities between TX9425 and Naso Nijo under the control conditions [33], while the differences between NILs were not significant in nonsalt grown plants (Figure 5). This indicates that analysis of physiological and molecular mechanisms involved in salinity tolerance is more reliable when using near-isogenic lines (NILs) with a common genetic background but contrasting levels of resistance to salt stress, as compared to comparing different genotypes.

### 3.2. The Effects of QSl.TxNn.2H on Biochemical Characteristics and Plant Ionome

Root Na^+^ content was not significantly different in roots of NIL, while its content in the leaves was 2-fold higher in the sensitive lines (Figure 3). This suggests that the delivery of xylem Na^+^ to the leaves was the major factor determining differential salinity tolerance in this pairs of NILs. HKT-mediated Na^+^ exclusion from the leaves represents a widely conserved primary salt tolerance mechanism in Arabidopsis and monocot crop plants [34]. Reduced leaf Na^+^ content may also be a result of more effective control of xylem Na^+^ loading. The latter process is considered to be thermodynamically active [35,36] and mediated by either SOS1 Na^+^/H^+^ exchangers that are preferentially expressed at the xylem symplast boundary of roots [37], or by the cation–Cl (CCC) cotransporters [38]. Thus, the QTL *QSl.TxNn.2H* is very likely a candidate for a major locus controlling xylem Na^+^ loading in barley.

The roles of proline and AO enzymes in the adaptation to saline environments is a matter of debate. While some studies have claimed that the accumulation of proline and higher AO enzyme activity play a protective function in plant defense responses against salt stress [39,40,41,42,43], others argued that they are merely stress “markers”, which play no causal role in plant adaptation to salinity [7,25,41,44]. In this study, a significant increase in antioxidative responses and a high proline accumulation were induced by salinity. The higher activities of AO enzyme at a specific time or tissue in tolerant lines can be detected under the background of salt tolerance QTL *QSl.TxNn.2H*, which may remove more ROS, thus reducing the accumulation of H_2_O_2_ and associated lipid peroxidation and the damage to the cell membrane. The remarkable increase in proline content in salinity-sensitive lines, however, indicates that it is a symptom of injury rather than an indicator of salinity tolerance. As for the changes in the AO enzyme activity, due to their dynamic nature and high tissue specificity, they are not suitable as biochemical indicators for the selection of salt tolerance genotypes [33,44,45], although their roles cannot be ignored under certain genetic backgrounds.

### 3.3. ROS Scavenging, Photosynthesis, and ATP Synthase Related Proteins Upregulated in Salinity Tolerant Lines and Underly the Tolerance Mechanism

Reactive oxygen species (ROS) are produced as a normal by-product of plant cellular metabolism by numerous processes, including photosynthesis and respiration. Under stress conditions, a dramatic increase in ROS production can be observed. An imbalance between ROS generation and ROS detoxification and excessive production of ROS are harmful for plants, and ultimately lead to cell death [46]. Significant increases in the abundance of ROS scavenging enzymes under salinity stress have been extensively reported [29,47,48,49]. In this study, corresponding to the biochemical results, lower MDA and H_2_O_2_ and higher AO enzyme activities in tolerant lines increased the abundance of proteins associated with ROS scavenging, such as peroxiredoxin-2 (spot 319) and superoxide dismutase [Cu-Zn] (spot 519) in leaves and the peroxidase superfamily proteins (spots 187, 102, 109, 167), and glutathione S-transferase family protein (spots 228, 245, 765) in roots were only shown in the salinity tolerant line (Figure 6, Table S1 and Table S2).

Photosynthesis is one of the fundamental biochemical processes that converts light into chemical energy which is severely affected by environmental stresses [50]. In the salinity tolerant line, increased abundance of photosynthesis-associated proteins, a PsbP family protein (spot 127), fructose-bisphosphate aldolase 2 (spots 251, 266), and the cytochrome b_6_-f complex iron-sulfur subunit (spot 528) were only observed under salt stress (Figure 6, Table S1). PsbP and PsbQ homologs are not only involved in PSII regulation and PSII repair, but also in chloroplast NDH activity and PSI assembly [51,52]. As the cytochrome b_6_-f complex determines the rate of electron transport through the electron transport chain and, concomitantly, the CO_2_ assimilation rate and the increased expression in salinity-stressed leaves, plants that are able to maintain the production of ATP and NADPH can provide energy for carbon reduction [53,54,55]. Fructose 1,6-biphosphate aldolase (FBA) is involved in glycolysis and gluconeogenesis in the cytoplasm and the Calvin cycle in plastids and poses great potential to control photosynthetic carbon flux and increases photosynthetic rate [56]. Fan and coauthors demonstrated that SpFBA plays very important roles in improving the survival ability of *S. portulacastrum* under high salinity conditions [57]. The overexpression of FBA in plastids enhanced the photosynthesis and growth of transgenic plants [58].

Energy (in the form of ATP) drives biosynthetic reactions in plant cells which are mainly produced by chloroplasts and mitochondria. When plants are exposed to salinity stress, energy requirements may increase considerably to operate several energy-consuming adaptive mechanisms including ion homeostasis, ROS defense, and osmotic adjustment [12,59]. Enhanced photosynthesis and redox energy production contribute to higher salinity tolerance [60]. Excess photosynthetic energy dissipation might play a role in defense photodamage, photoinhibition, and photo-oxidative salinity tolerance [61]. The enhanced NDH-dependent CEF around PSI supplies extra ATP which sequester Na^+^ into the vacuole, thus alleviating the damage to the photosynthetic apparatus and conferring plants salinity tolerance [62]. The RMtATP6 protein acts as a subunit of ATP synthase in mitochondria, and is induced under salinity stress and the overexpression of RMtATP6; it also provides greater tolerance to salt stress in tobacco. Increased abundance of three proteins associated with ATP production, such as ATP synthase subunit alpha (spot 36), ATP synthase D chain (spot 322) and ATP synthase subunit delta (spot 287) were only observed in the salinity tolerant line under salt stress (Figure 6, Table S2), indicating that the synthesis of ATP in the salt tolerant line might be enhanced. Overall, under stress conditions, the increased abundance of photosynthesis, ROS scavenging, and ATP synthase associated proteins in the tolerant line may play important roles in protecting photosystems machinery, alleviating oxidative stress and providing additional energy needed for cell homeostasis.

In conclusion, the QTL *QSl.TxNn.2H* could improve salinity tolerance by controlling Na^+^ loading into the xylem to reduce Na^+^ toxicity in leaves and inducing upregulation expression of proteins related to photosynthesis, ROS scavenging, and ATP synthase genes to protect the photosynthetic apparatus, thus alleviating oxidative stress and providing additional energy.

## 4. Materials and Methods

### 4.1. DNA Extraction and Genotype Screening

DNA of NILs was extracted from the leaf tissue of four-week old seedlings, according to the plant DNA extraction protocol for DArT analysis (https://www.diversityarrays.com/files/DArT_DNA_isolation.pdf). The two parental cultivars and NILs were genotyped with DArTseq (http://www.diversityarrays.com/dart-application-dartseq). Around 10,000 polymorphism molecular markers with known positions were chosen to compare the differences between NILs and relationships to their parents.

### 4.2. Development of Near Isogenic Lines (NILs)

A DH population was constructed from the cross of TX9425 and Naso Nijo [26]. Two pairs of DH lines with similar agronomic traits but contrasting salinity tolerance were selected for crossing. Heterozygotes in the QTL region were screened for by the nearest marker bpb-6792 from F_2_ to F_5_ through marker assisted selection. In F_6_ plants, by screening phenotype (320 mM NaCl treatment at the two and a half leaf stage) and genotype (DArTseq analysis) to select two pairs of near-isogenic lines (N33 and T46, N53 and T66). T46 and T66 have a homozygous salt tolerance allele from TX9425, and N33 and N53 have a homozygous salt sensitive allele from Naso Nijo (Figure 1A,B).

### 4.3. Growth Condition and Materials Collection

Two pairs of near-isogenic lines (N33 and T46, N53 and T66) containing the major QTL for salt tolerance (*QSL.TxNn2H*) were grown in a glasshouse at Yangzhou University, Jiangsu, China. Plants were grown in 2L containers (one pair of NILs per container, each line contains three plants) filled with the standard fertilized potting mix. On day 20 of normal growth (three leaf stage), a salt stress treatment was applied by adding 300 mM NaCl, according to our previous method [26]. After 0, 2, 4, and 6 days, roots and the top three leaves of two pairs of NILs were washed with distilled water to remove soil and other contaminants and snap frozen in liquid nitrogen for the measurement of physiological characteristics. After 0 and 4 days, the roots and leaves of one pair of NILs (N33 and T46) were collected for protein and RNA analysis. Three independent replications were used for every experiment.

### 4.4. Determination of H_2_O_2_, MDA and Proline Measurements Contents

H_2_O_2_ content was measured by a modified method of Ghiazdowska et al. [63]. The frozen samples (0.5 g) were homogenized in an ice bath with 3 mL of 0.1% (*w/v*) trichloroacetic acid (TCA), and centrifuged at 12000 *g* for 15 min at 4 °C, and 0.5 mL of the supernatant was collected and added to 0.5 mL of potassium phosphate buffer (10 mM, pH 7.0) and 1 mL of potassium iodide (1 M). The H_2_O_2_ content was measured at 390 nm. H_2_O_2_ standards ranging from 10 to 100 μM were used to prepare a standard curve for estimating chlorophyll content in the samples.

Malondialdehyde (MDA) contents were assayed by the method of Dionisio-Sese and Tobita [64]. The frozen samples (0.5 g) were homogenized in 5 mL of phosphate butter (0.05 mol, pH 7.8, 1% polyvinylpyrrolidone contained). The extract was centrifuged at 4 °C for 10 min at 12,000 *g*, and 1 mL of the supernatant was mixed with 1 mL of 0.5% (*w/v*) thiobarbituric acid solution (containing 20% trichloroacetic acid) and heated at 95 °C for 30 min. The reaction was stopped in an ice bath. The mixture was centrifuged at 4 °C for 10 min at 10,000 *g* and the absorption of the supernatant measured at 532 nm and 600 nm.

Proline concentration was quantified using a modified method described by Bates et al. [65]. Approximately 0.5 g samples were homogenized in 10mL of 3% (*w/v*) sulfosalicylic acid. A total of 2 mL of filtrate, 2 mL of glacial acetic acid, and 3 mL of acid-ninhindrin were heated for 1 h at 100 °C. After cooling, 5 mL toluene was added and then placed in the dark for 30 min. The absorbance value was determined at 520 nm. The L-proline concentration was determined using a standard curve ranging from 10–100 g/mL.

### 4.5. Antioxidant Enzyme Activity Measurements

Fresh samples (0.1 g) were ground in liquid nitrogen and then suspended in 0.9 mL phosphate buffer (10 mM, pH 7.4). The homogenate was centrifuged at 4 °C and 2500 rpm for 10 min, and the resulting supernatant was collected. The activities of superoxide dismutase (SOD, A001-3-2), catalase (CAT, A007-1-1), peroxidase (POD, A084-3-1), ascorbate peroxidase (APX, A123-1-1), and glutathion reductases (GR, A062-1-1) were measured using commercially available kits from Nanjing Jiancheng Bioengineering Institute (Nanjing, China) following the manufacturer’s instructions.

### 4.6. Na^+^ and K^+^ Content

Dried materials (0.1 g) were ground into fine powder and digested in concentrated sulfuric acid at 370 °C for 20 min. A few drops of 30% hydrogen peroxide were added until the solution became clear and transparent. The Na^+^ and K^+^ concentrations were determined by a flame photometer (Model 420, Sherwood, Cambridge, UK).

### 4.7. Protein Extraction, 2-DE, and Image Analysis

TRIZOL^®^ reagent (Invitrogen, USA) was used to extract total protein according to Jaipal with some modifications [66]. Briefly, the frozen samples (0.5–1 g) were homogenized under liquid nitrogen to a fine powder. A total of 1 mL of TRIzol reagent and 200 uL of chloroform were added, mixed vigorously for 15 min and incubated at 25 °C for 5 min followed by centrifugation at 12,000 *g* for 15 min at 4 °C. The lower organic phase was moved to a new 1.5-mL tube and mixed with 300 uL ethanol and 1 mL ice-cold acetone to precipitate the protein. The protein pellet was thoroughly washed with 0.3 M guanidium-HCl in 95% ethanol three times, followed by ice-cold acetone twice. After air-drying, all protein pellets were dissolved in rehydration buffer [7-8 M urea, 2 M thiourea, 2–4% CHAPS, 1% IPG buffer and BPB]. Protein quantification was determined via the Bradford assay with bovine serum albumin (BSA) as a standard. Protein (350 ug) was loaded on 24 cm IPG strip with a linear gradient (pH 4–7), and SDS-PAGE was performed with 12.5% gel. Proteins were visualized by silver staining. Then, 2-DE Images were obtained by scanning each stained gel at 600 dpi resolution using an ImageScanner and analyzed using The Imagemaster 2D Platinum Software Version 7.0 (GE Healthcare). Protein spots with more than a 1.5-fold changes among the treatments and significant at *p* < 0.05 were considered to be differentially expressed spots.

### 4.8. Identification of Proteins by Mass Spectrometry (MS)

The differentially expressed protein spots were excised from the 2-DE gels and digested with trypsin. Proteins were identified using SCIEX MALDI TOF-TOF™5800 analyzer equipped with neodymium. Combined MS and MS/MS results were analyzed using ProteinPilot software (Foster City, CA, USA) and searched using MASCOT software (http://www.matrixscience.com/). The criteria for selection of the matched protein sequence followed by method of Guo [67].

### 4.9. Statistical Analysis

Statistical analysis was performed with SPSS Statistics 19.0 software. One-way analysis of variance (ANOVA) was used to evaluate the significance of differences between the exposure groups and the control. Experimental data was reported as the mean ± standard deviation. The Student’s *t*-test was used to evaluate the significance of the differences among NILs. Significant differences were indicated by asterisks (*p* < 0.05).

## Supplementary Materials

Supplementary materials can be found at https://www.mdpi.com/1422-0067/21/4/1516/s1.
